# A Cross-Cultural Comparison on Implicit and Explicit Attitudes Towards Artificial Agents

**DOI:** 10.1007/s12369-022-00917-7

**Published:** 2022-09-28

**Authors:** Fabiola Diana, Misako Kawahara, Isabella Saccardi, Ruud Hortensius, Akihiro Tanaka, Mariska E. Kret

**Affiliations:** 1 Comparative Psychology and Affective Neuroscience Lab, Cognitive Psychology Unit, Leiden University, Wassenaarseweg 52, 2333 AK, Leiden, The Netherlands; 2 Leiden Institute for Brain and Cognition (LIBC), Leiden University, Albinusdreef 2, 2333 ZA, Leiden, The Netherlands; 3 Department of Psychology, Tokyo Woman’s Christian University, 2-6-1 Zempukuji, Suginamiku, Tokyo 167-8585 Japan; 4 Department of Information and Computing Sciences, Utrecht University, Princeton Square 5, 3584 CC Utrecht, The Netherlands; 5 Department of Psychology, Utrecht University, Heidelberglaan 1, 3584 CS Utrecht, The Netherlands

**Keywords:** Implicit attitudes, Explicit attitudes, Human–robot interaction, Cross-cultural, Human-like robots, Machine-like robots

## Abstract

Historically, there has been a great deal of confusion in the literature regarding cross-cultural differences in attitudes towards artificial agents and preferences for their physical appearance. Previous studies have almost exclusively assessed attitudes using self-report measures (i.e., questionnaires). In the present study, we sought to expand our knowledge on the influence of cultural background on explicit *and* implicit attitudes towards robots and avatars. Using the Negative Attitudes Towards Robots Scale and the Implicit Association Test in a Japanese and Dutch sample, we investigated the effect of culture and robots’ body types on explicit *and* implicit attitudes across two experiments (total *n* = 669). Partly overlapping with our hypothesis, we found that Japanese individuals had a more positive explicit attitude towards robots compared to Dutch individuals, but no evidence of such a difference was found at the implicit level. As predicted, the implicit preference towards humans was moderate in both cultural groups, but in contrast to what we expected, neither culture nor robot embodiment influenced this preference. These results suggest that only at the explicit but not implicit level, cultural differences appear in attitudes towards robots.

**Supplementary Information:**

The online version contains supplementary material available at 10.1007/s12369-022-00917-7.

## Introduction

When introducing artificial agents into an intimate environment, such as in households or schools, the acceptance by the users is indispensable [1]. Over the last few decades, human–robot interaction (HRI) research is facing significant challenges in terms of the social acceptance of robots and virtual agents [2]. The most plausible explanation for this impasse lies in the interdependence between the social acceptance of artificial agents and the users’ attitudes towards those agents [3]. Human attitudes towards artificial agents are influenced by several factors, including users’ cultural background and agents’ embodiment [4], ultimately impacting quality, engagement, and effectiveness of the interaction [5, 6]. Despite the voluminous literature on cross-cultural differences in the attitude towards artificial agents [7], and on the preferences towards a specific agent’s embodiment [8], the results are substantially mixed so far. In the majority of these experiments, attitudes are assessed using self-report measures (i.e., questionnaires). This approach is limited as it is not always a reliable measure of people’s actual thoughts [9]. Therefore, the goal of this research was twofold: (i) to offer additional insights into the effect of agents' embodiment and individuals’ cultural background on the attitude towards robots and avatars and, within this framework, (ii) to compare implicit and explicit measures to assess human attitudes towards artificial agents.

Perception is intimately related to attitudes. Through perception, we can understand and organize sensations into a meaningful experience, and this interpretation may determine our attitudes and (future) actions towards that experience [10]. A growing body of research on HRI suggests that our perception of embodied and virtual agents hinges on a plethora of factors: some are akin to the agent itself, such as its physical appearance, social skills, behavior, and context of use [11]; others, are utterly subjective human or cognitive factors such as beliefs and prior experience that contribute shaping our perception in general [12]. Specifically while interacting with embodied or virtual machines, expectations seem to play a decisive role in shaping our perception [12, 13, 14, 15]. Humans are generally proficient at a wide-ranging batch of capabilities. In contrast, robots and virtual avatars are conceived to be skilled at a few limited capabilities related to a specific context, even though humans might perceive them as more intelligent than they actually are. This discrepancy is already enough to generate an *expectation gap*, a phenomenon that occurs when humans engage with a complex engineered system and develop expectations that are misaligned with the system’s capabilities [5, 6, 16]. From a design perspective, the aim should be to have a neutral or positive gap, and the agent’s performance should meet or exceed users' expectations [17]. While high reliability of the agent is associated with higher trust [18], unmet expectations can lead to users’ disappointment, and eventually distrust towards the agent [13, 19]. Not correcting this expectation gap could result in less effective human–machine cooperation, along with less efficiency in improving the agent behavior within its specific context of use [16].

Besides expectations, stimulus factors such as an agent’s embodiment can underlie an overestimation of the agent’s capabilities. For instance, if an agent has a human-like appearance, it is easier to misattribute human mental models to agents, overdrawing the range of tasks that they can truly perform [5, 6, 16]. This perceptual mismatch can result in users’ disappointment [16]. It is not yet clear whether one particular agent’s body type is preferable to another: on the one hand, humanoid agents could trigger more empathetic responses based on physical similarities [20]; on the other hand, less human-realistic agents might reduce eeriness and increase trust [21]. Research on this subject has reported heterogeneous outcomes. Some studies have found generic positive attitudes towards human-like robots. Participants, including older adults, expressed a significant social acceptance of humanoid robots [22, 23], considering them more conscientious and intelligent [8]. Furthermore, human-like robots tend to be perceived as less threatening by adults, and they are treated more politely compared to machine-like robots [24–26]. Comparable results have been found also for virtual avatars, as human-realistic avatars elicited greater affinity and preference than cartoon-like avatars [27]. On the other hand, quite a few studies support the opposite position. A significant preference for a small machine-like robot, compared to humanoid and zoomorphic, has been demonstrated also in the adult population [28]. In addition, machine-like robots seem to be preferred in terms of initiating contact, emotional connection, meeting expectations, and appeal [29]. A study by Wood found that children perceived humanoid robots as more aggressive and threatening, while machine-like robots are considered friendlier [30]. Studies on virtual agents found distinct preferences toward cartoon-like avatars compared to human-realistic ones [31]. It is important to mention that, along with opposite positions, there is also a middle ground. In fact, not all studies found significant differences between diverse shapes of robot bodies [32] and if they did, they were not always extensible to all domains investigated by the authors [24].

One possible explanation of these contradicting findings lies in the variety of factors that influence that preference, in particular the cultural background of the perceiver [33]. A cultural lens has been widely adopted in human–robot interaction research (for a review, see [7]). Based on the work by Lim and colleagues, we operationalize the cultural background as a national culture—values, norms, and practices that are undertaken by the people born and raised in a certain country [7]. Among all the cultures that have been compared on this topic, the Japanese culture has been investigated the most. This is likely given the decisive contribution that Japan has made in social robotic research, along with the large usage and promotion of social robots from the government [34]. Today, according to the International Federation of Robotics report (2021), Japan’s manufacturers deliver 45% of the global robot supply and robotics represents a fundamental branch of the Japanese economy. Consequently, robots in Japan have historically had a different media exposure, often portrayed as heroic and righteous (

e.g., Doraemon, Astro Boy), compared to Europe. A popular explanation of this positive view is that, in the traditional Shintoism religion, inanimate objects are recognized as possessing a soul [35]. Some authors have suggested that such a philosophical orientation might lead to more willingness to accept artificial agents into society and this, along with the above arguments, led to the erroneous speculation that Japanese culture has generally a positive attitude towards artificial agents compared to other cultures, the so-called Japanese “Robot Mania” [36]. The shreds of evidence supporting this phenomenon are discordant. Several studies found results in line with this assertion. Nomura and colleagues found Japanese participants to have a positive attitude towards robots compared to UK participants (but there is an effect of age [37]), and Korean participants [38], and were more inclined to accept robots doing social activities and to have warmer feelings towards them [34, 36, 38]. Along with the arguments pro-Japanese “robophilia”, a considerable body of literature found results in the opposite direction. In a cross-cultural investigation, Bartneck and colleagues revealed that Japanese individuals were far more concerned about the social impact of robots, compared to US and Dutch individuals [39]. In a follow-up study by the same authors, Japanese participants, while not concerned about the social impact, reported concerns about the emotional aspect of interacting with robots [40]. Other studies show a similar variety in results. Japanese participants expressed more negative feelings towards robots compared to participants in the UK, while at the same time, they were also more prone to let the robots perform tasks that required human characteristics [41]. It has also been shown that Japanese participants have less robot acceptance and trust compared to German participants, even though they both showed similar levels of anxiety and perceived agency [42]. Taken together, studies have reported both positive and negative attitudes towards robots across dimensions of perception and interaction. These findings harmonize well with the considerations by Bartneck and colleagues [39]. They propose that the same over-exposure used to explain positive attitudes towards robots in Japan can simultaneously be the main cause of their concerns, due to a more realistic view of the assets and limitations of this new technology.

Similar controversial results can be found while investigating cultural preference towards a *specific* robot body. It has been shown that Japanese participants have a preference towards human-like robots [37, 39], and they feel more comfortable with technology behaving like humans compared to US participants [43, 44]. Other works found a reversed trend: the more robots were human-like, the less Japanese individuals liked them [45]. It is undeniable that the human–robot interaction literature tended to focus on Japan over other countries in cross-cultural studies. A positive fallout is the enormous amount of information we have on Japanese attitudes towards artificial agents, which is perfect for comparisons. Nonetheless, this has also led to neglecting other countries (e.g., European) in which the integration of social robots and their social acceptance is no less urgent. For instance, only one study has compared the Dutch population to other countries in terms of attitudes towards artificial agents [39], suggesting that Dutch participants have a more negative attitude towards the impact that robots might have on society compared to Japanese.

The main point that emerges from the research on cultural similarities and differences in the attitude towards artificial agents is the significant fragmentation of the literature. Common ground among all these different studies is the type of instrument used to measure those variables. In fact, with only a few exceptions, the majority of the studies employed the Negative Attitude Towards Robots Scale (NARS) [46]. This questionnaire consists of three parts: attitudes toward the interaction with robots; attitudes toward the social influence of robots; and attitudes related to emotions felt during interaction with robots. The NARS is a self-report measure, and unfortunately, such *explicit* measures of attitudes are vulnerable to two kinds of biases. First, participants might be unaware of attitudes influencing their behavior and, if unsure, they might answer based on the most popular view [36]. Second, if participants are aware of the attitudes behind their behaviors, they could consciously decide to disguise them. Sometimes this is the reflection of a desire to conform with others, which could lead to self-presentational and social desirability biases [9]. A solution to avoid these complications is to make use of *implicit* measures. These types of measures are more predictive of behavior than explicit ones [47]. To date, there is no recognized implicit measure of attitudes towards artificial agents. Nevertheless, numerous attempts have been made in the literature. In their recent paper, Marchesi and colleagues used the cyber ball task to investigate cross-cultural differences in the social exclusion of social robots [48]. In addition, some authors developed a measure of semantic priming to evaluate whether participants consider humans and robots as similar or different [49]. Others used observable behavior, such as the distance participants were maintaining between themselves and the agents [50]. A problem with this approach is that behavior may be biased by the context of interaction (e.g., experimental setting, task). While incorporating the critical factor of physical embodiment [51], cross-platform generalizability [52] is more difficult to achieve. A method that merges the generalizability of the self-report with the power to assess implicit attitude is the Implicit Association Tests (IAT) [9]. The IAT measures association between two target concepts (e.g., *human* and *robot*) along an attribute dimension (e.g., *good* and *bad*) based on the latency of the responses. A positive implicit attitude towards a target is reflected in shorter latencies in associating positive attributes to the construct. Vice versa, the more negatively a target is perceived, the longer the latency will be in associating the target with positive attributes. The idea is that responding is easier when closely related items share the same response key (e.g., robot-bad). To the best of our knowledge, only two studies used a combination of explicit measures and IAT in research on attitudes towards artificial agents and found contrasting results across these measures. Sanders and colleagues have found more positive implicit attitudes towards humans compared to robots, along with a lack of correlation between implicit and explicit measures [53]. Due to the limited number of studies, it is unknown if implicit attitudes towards robots are cultural-independent. One study by MacDormand and colleagues found that Japanese individuals have warmer (explicit) feelings towards robots compared to US participants, but the IAT (implicit) revealed a similar human preference across both cultures [36].

Here, we aim to fill the gaps in the literature by teasing apart factors that contribute to shaping our attitudes towards artificial agents, specifically cultural background and agents’ body type. To consider the multiple dimensions that attitudes encompass, we assessed both implicit and explicit attitudes towards artificial agents across two cultural groups (Dutch and Japanese individuals) in two experiments. Participants were asked to complete two online Implicit Association Test tasks and to fill in the Negative Attitude towards Robots Scale (NARS) questionnaires. The first goal of the research was to investigate the cross-cultural differences in implicit and explicit attitudes towards artificial agents. The majority of the studies on the Japanese sample found a negative attitude towards robots only for *specific* subscales of the NARS, but always a different one [social—36], [emotional—48]. Given this observation, we hypothesized that Japanese compared to Dutch participants will have a more positive general explicit attitude towards robots and avatars, expressed by a lower general NARS score (H1). The second goal of our study was to investigate the cross-cultural effect of agents’ appearance on implicit attitudes towards robots and avatars. Based on the reported implicit preference for humans compared to robots and the potential cultural independence of this effect [36, 53], we expected to find a significant preference towards humans in general, expressed by a D-score > 0.15 (H2a). Given the great exposure that robots have in Japanese society, we still expected differences in the implicit attitude towards robots between the two cultures. We hypothesized that Japanese participants, while having an implicit preference for humans over robots, would have a more positive implicit attitude towards robots and avatars, compared to Dutch participants, expressed by a larger positive D-score for Dutch compared to Japanese participants (H2b). As a preference toward non-realistic human-like robots was not influenced by culture [32], we expected that participants would have a generally more positive implicit attitude towards human-like robots compared to machine-like robots regardless of the culture (H3). While results have shown a preference towards machine-like robots when comparing these with hyper-realistic humanoid robots [29, 45], we used human-like robots in the present study, which differentiate themselves from machine-like robots merely by humanoid features such as familiar body shape, eyes, and head.

## Method

### Data Statement

Data, materials, and code are publicly available on the OSF at this link https://osf.io/uat6r/. We report all measures in the study, all manipulations, any data exclusions, and the sample size determination rule.

### Participants

We recruited 669 participants (280 Dutch, 389 Japanese), for two online experiments via the open-source platform Jatos [54]. We aimed to recruit a total of 160 participants for each experiment. The online recruitment was coordinated by Leiden University (NL) in collaboration with Tokyo Woman’s Christian University (JP), via the SONA system, Amazon Mechanical-Turk [29], Prolific (www.prolific.com), and CrowdWork [30]. For Experiment 1, participants were aged between 17 and 30 (Dutch, 63 women) and 18 and 30 (Japanese, 42 women). For Experiment 2, participants were aged between 35 and 58 (Dutch, 33 women) and 21 and 65 (Japanese, 41 women). Supplementary Table 1 provides further demographic details. The following inclusion criteria were used: Japanese or Dutch, with normal or correct-to-normal vision (glasses, contact lenses), no current or previous history of neurological and psychological impairment, and no regular use of psychoactive drugs. The experimental procedures of this study were approved by the Psychology Research Ethics Committee (2020-04-28-V2-2399). Before the experiment, written informed consent was signed online. Compensation consists of one credit or 2 euros (300 JPY). Among the 669 participants recruited, 320 participants completed both the NARS and the IAT tasks (Experiment 1: 80 Dutch, 84 Japanese; Experiment 2: 78 Dutch, 78 Japanese). Due to problems with the recruitment platform, 66 participants (56 Dutch, 10 Japanese) were not able to access the first questionnaire after signing the informed consent. Additionally, 11 participants did not report their demographic (6 Dutch, 5 Japanese), and 270 participants were excluded because of incomplete data due to various reasons (e.g., technical problems, termination before the end of the experiment (60 Dutch, 210 Japanese)).^1^ Before the data analysis, 2 Japanese participants were excluded based on the IAT exclusion criteria [55], as they had less than 3 correct responses with latency between 400 ms and 10 s in each block.

### Design

A two-sessions counter-balanced mixed design was used with participants’ nationality (Dutch, Japanese) as between factor and NARS subscale (interactions, emotions, social influence), IAT version (version 1: robots, humans; version 2: avatars, humans) as within factor. The order of sessions was counterbalanced, and the stimuli presentation was fully randomized. All tasks were done back-to-back, there was no time in between the NARS and IAT tasks. The dependent variables were the NARS score (explicit attitude) and the D-score (implicit attitude). Since the IAT has been suggested to be affected by a familiarity bias towards same-culture faces [56], we controlled for this by presenting in Experiment 1 faces that were representative of the culture (Dutch faces to Dutch sample; Japanese faces to Japanese sample), and in Experiment 2 faces that were not representative of the cultural background (Japanese faces to Dutch sample; Dutch faces to Japanese sample) and therefore less familiar.

### Materials

#### Negative Attitude Towards Robots (NARS)

The Negative Attitude Towards Robots Scale (NARS) [46] has been developed to measure humans’ attitudes toward communication with robots in daily life. It is composed of 14 items in total, measuring attitudes on three different subscales: (i) negative attitude in situations of interactions with robots (i.e., “*I would feel nervous operating a robot in front of other people*”); (ii) negative attitude towards the social influence of robots (i.e., “*I am concerned that robots would be a bad influence on children*”); (iii) negative attitude towards emotions in interaction with robots (i.e., “*I feel comforted being with robots that have emotions*”). Following the guidelines of Flora (2020), we estimated the reliability of the questionnaire calculating McDonald’s Omega [57]. The ωu-coefficients for the NARS subscales for the Dutch sample were 0.711, 0.694, and 0.643 (Experiment 1) and 0.683, 0.681, and 0.784 (Experiment 2) for Subscale 1, 2, and 3 respectively. The ωu-coefficients for the NARS subscales for the Japanese sample were 0.868, 0.859, and 0.807 (Experiment 1) and 0.703, 0.760, and 0.625 (Experiment 2) for Subscale 1, 2, and 3 respectively. See Supplementary material for details on the computation. Participants were asked to fill each item based on a 5-point Likert scale (1—Strongly disagree, 5—Strongly agree). As this is a self-report measure, we decided to treat it as an explicit measure of attitude. The original version of the NARS is in Japanese and has been validated in English [58]. Dutch participants performed the NARS in Dutch.

#### Implicit Association Test (IAT)

The Implicit Association Test (IAT) [9] measures the strength of association between concepts (e.g., human, robots) and evaluations (e.g., good, bad) or stereotypes (e.g., athletic, villain). The IAT has a total of 7-blocks. In IAT participants are asked to quickly sort words into categories that are on the right and left-hand side of the computer screen by pressing the “e” key if the word/picture belongs to the category on the left and the “i” key if belongs to the category on the right. The first two blocks (20 trials each) are practice blocks where participants sort pictures (robots, humans) into categories, followed by sorting words (Good, Bad) in the same categories (Supplementary Fig. 1). In the third block (20 trials) of IAT, categories are combined, and participants are asked to sort both concept and evaluation words. For instance, for the categories “Robot-Bad” and “Human-Good”. The fourth block (20 trials) is the same as the previous block but with 40 trials instead. The fifth block (20 trials) is the same as block one, but the positions of the two categories are reversed. The sixth block (20 trials) is the same as the third, but with inverted categories (“Robot-Good”, “Human-Bad”). Lastly, the seventh block is the same as the previous one but with 40 trials. The side the category is presented (left or right) is counterbalanced (“Good” can be either on the left or right). The IAT measures response accuracy and reaction times. The overall duration of the task is around 6 min. The IAT was built in OpenSesame 3.2.8 [59], conducted on the online platform JATOS—Just Another Tool for Online Research [54], and translated into Japanese or Dutch.

### Stimuli

#### Robot

**Fig. 1:**
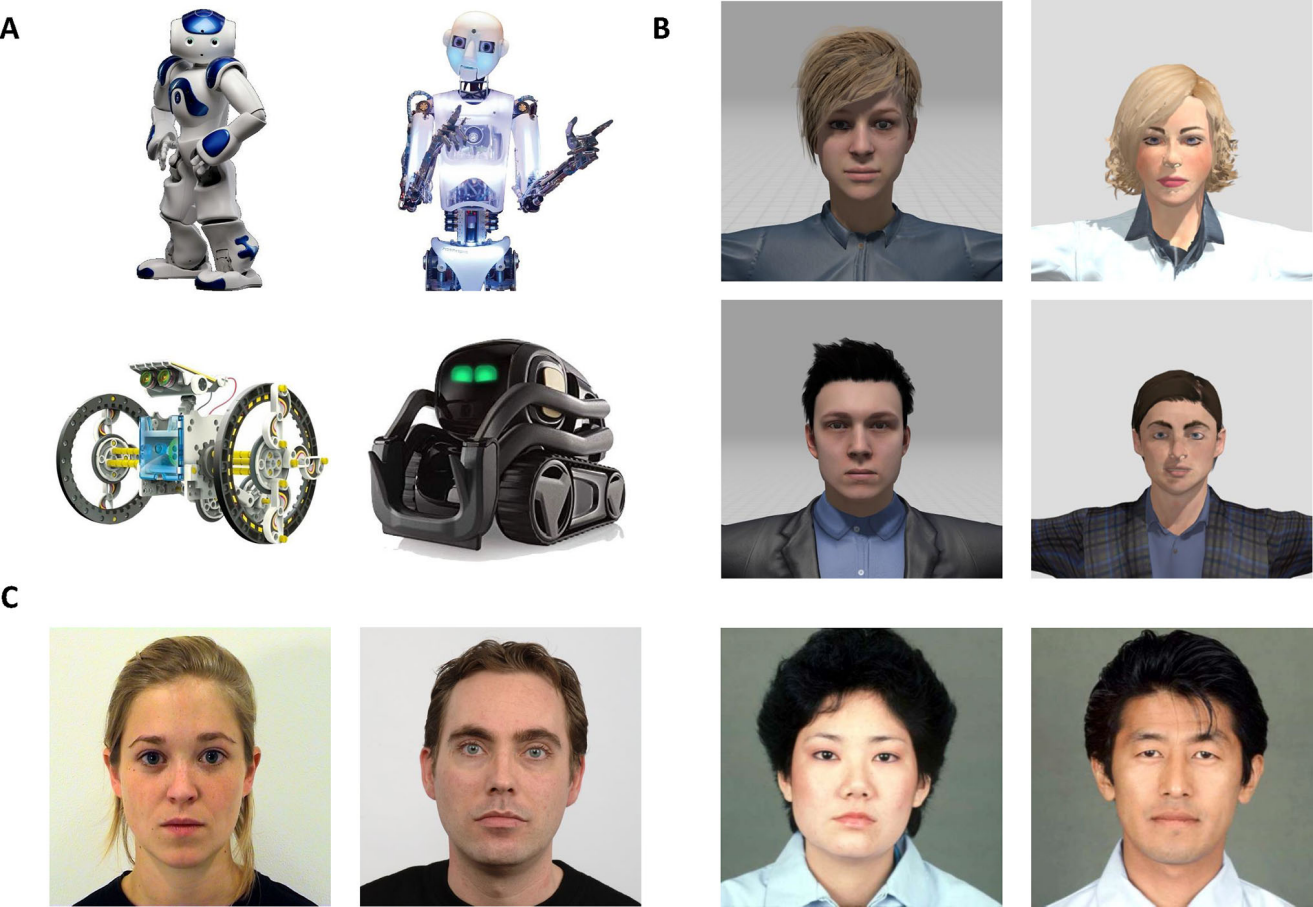
**A** Example pictures for the IAT version 1 (robots). In the upper line, human-like robots, and in the bottom line examples of machine-like robots; **B** Example pictures for the IAT version 2 (avatars); **C** Examples of the human pictures used in both IAT versions. On the left, examples of pictures presented to the Dutch participants. On the right, example of pictures presented to the Japanese participants

In the first version of the IAT, we used pictures of robots that are currently commercially available on the market: 15 images depict anthropomorphic human-like robots, with familiar bodies and faces aiming to reproduce a human shape, while 15 represent machine-like robots, without a particular shape that could remind a human or an animal (Fig. 1). The human-likeness of the robot was taken from the ABOT database (http://www.abotdatabase.info/; Supplementary Table 2) [60]. The images were pre-processed to have a dimension of 500 × 500 pixels, equal contrasts, and equal luminance. Since the robot pictures have far more variance than human faces (for shape and colors), we opted for three different versions of the Robots IAT. In each version, 10 images were randomly chosen (5 human-like, 5 machine-like). The three different versions were counterbalanced across participants.

#### Avatars

In the second version of the IAT, we used two-dimensional avatars based on a 3D human model, downloaded from the Mixamo (Adobe Systems Incorporated, 2020) and Sketchfab (Sketchfab Incorporated, 2020). We downloaded 10 images depicting avatars from the bust to the face (5 Females, 5 Males, Fig. 1). Pictures were pre-processed and cut with the GNU Image Manipulation Program (GIMP Development Team, 2020) into images of 252 × 319 pixels, equalized for luminance and contrast.

#### Human Faces

The human faces displayed in both sessions of the IAT were chosen from two standardized datasets. We opted to include only neutral facial expressions to avoid association biases due to the emotional valence of the pictures (see Fig. 1). For Experiment 1, in the Dutch IAT experiment, we randomly selected 10 pictures (5 Females, 5 Males) from the Radboud Faces Database [61] and the Amsterdam Dynamic Facial Expressions Set (ADFES) [62]. In the Japanese IAT version, we randomly selected 10 pictures (5 Females, 5 Males) from the Japanese and Caucasian Neutral Faces (JACNeuF) [63]. Crucially, in Experiment 1 we presented Dutch faces and Japanese faces to Dutch and Japanese samples respectively, while in Experiment 2 we presented Japanese and Dutch faces to Dutch and Japanese samples respectively.

### Procedure

The procedure was the same for the Dutch and Japanese participants. The experiment started from the recruitment platforms, Amazon’s Mechanical Turk, Prolific, and SONA for Dutch participants, and Crowdwork for Japanese participants. From these platforms, they were redirected to Qualtrics to read the information letter, provide consent, and complete the demographic questionnaire (age, gender, education level, nationality, and email address on which they received a link to the Jatos experimental framework). After the demographic questions, the NARS questionnaire was administered. We opted to include the NARS before the IAT because we wanted the participants to fill out the questionnaire and report their explicit attitudes based on their mental idea of the robot. Including the NARS after the IAT would likely bias participants toward a specific robot’s shape. Following this, participants were redirected to the JATOS web server to start the IAT task. The total time of completion for the study was around 15 min. Before each subsequent block iteration, participants were informed about what key input (“e” or “i”) represented what attribute and/or concept. Upon completion of the first IAT version, participants started the IAT task with the second version (either robots or avatars versus humans). When the participants completed both IAT versions, the webserver redirected them to the final Qualtrics survey, where participants were debriefed.

### Data processing and analyses

For the NARS questionnaire, the items were averaged for each subscale reflecting explicit attitudes towards interactions (subscale 1), emotions (subscale 2), and social influence (subscale 3). Higher scores reflect a stronger negative explicit attitude towards robots. For the IAT task, we calculated the D-score which references an implicit preference toward one category over the other category [9]. The D-score is the average difference in response latency between combined tasks divided by the inclusive standard deviation [9]. A larger positive D-score represents a less favorable implicit attitude towards robots compared to humans. The D-score ranges from − 2 to 2, with  − 2 being total preference toward robots (version 1) or avatars (version 2), and 2 being total preference toward humans (both versions). A value of around − 0.15/ 0.15 would indicate a light preference, a value of around − 0.35/0.35 a moderate preference, while a value of around − 0.65/0.65 indicates a strong preference for the specific category. No preference for either humans or robots would be concluded if the score fell within a range of − 0.15 and 0.15. We calculated 4 D-scores in total: D-score for the IAT version 1 (robot), D-score for the IAT version 2 (avatar), and two D-score for the machine-like and human-like robots, both based on IAT version 1. We excluded RTs based on the criteria of Greenwald and colleagues (2003, p.214, Table 4). We excluded trials with latencies < 400 ms. In addition, the D algorithm (IATscores package) excludes trials with latencies > 10 s by default. Participants with less than 3 correct responses with latency between 400 ms and 10 s in each block are excluded from the analyses, as short response times could indicate deceptive responses [55]. Participants that did not complete all blocks of the IAT version were not included in the sample. The D-score was calculated with R library *IATscores,* using R v4.1.2 (R Core Team, 2016).

All statistical analyses were performed using JASP v0.16 (JASP Team, 2021). All the variables' distribution met the assumptions of normality (Shapiro–Wilk tests) and homogeneity of the variance (Levene’s test). To test the hypotheses, we conducted a series of Repeated-Measure ANOVA to analyze the explicit attitudes toward robots (H1) and the implicit attitudes towards robots and avatars (H2a and H2b), and towards specific robots' body types (machine-like, human-like, H3). In Experiment 1, age between Dutch sample (Mean = 19.75, SD = 2.38) and the Japanese sample (Mean = 25.88, SD = 3.60) differed significantly (t =  − 12.60, *p* =  < 0.001, MD =  − 6.131, 95% CI [ − 2.34,  − 1.59]). We controlled for age twofold. We performed linear regression with age as covariate and Nationality as a factor to predict the dependent variable central to the hypothesis (e.g., D-scores of Robot IAT). After excluding the presence of multicollinearity issues, age was included as a covariate in the Repeated Measures ANOVAs. Similarly, the level of Education, re-coded based on the UNESCO International Standard Classification of Education, was included as a covariate in the main analyses for Experiment 1. No significant effect of age and level of education on the explicit nor implicit attitude towards robots was found (Supplemental Material). For Experiment 1 we conducted a total of three Repeated Measures ANOVA (rmANOVA): to test H1, we conducted a rmANOVA with NARS score as a 3-level factor (Subscale 1, Subscale 2, Subscale 3), Nationality as a between-subject factor, and Age and Level of Education as covariates; to test H2a, we conducted two one-sample t-tests (test value = 0.15), one for each nationality with the D-scores of Robot IAT and Avatar IAT as variables. To test H2b, we conducted a rmANOVA with D-score as a 2-level factor (Avatar IAT, Robot IAT), Nationality as a between-subject factor, and Age and Level of Education as covariates; to test H3, we conducted a rmANOVA with Robot IAT D-score as a 2-level factor (machine-like, human-like), Nationality as a between-subject factor, and Age and Level of Education as covariates. In Experiment 2, age between Dutch sample (Mean = 42.65, SD = 5.85) and Japanese sample (Mean = 41,29, SD = 9,60) did not significantly differ (*p* = 0.288). For Experiment 2, we conducted three rmANOVA: to test H1, we conducted a rmANOVA with NARS score as a 3-level factor (Subscale 1, Subscale 2, Subscale 3) and Nationality as a between-subject factor; to test H2a, we conducted two one-sample t-tests (test value = 0.15), one for each nationality with the D-scores of Robot IAT and Avatar IAT as variables. To test H2b, we conducted a rmANOVA with D-score as a 2-level factor (Avatar IAT, Robot IAT) and Nationality as a between-subject factor; to test H3, we conducted a rmANOVA with Robot IAT D-score as a 2-level factor (machine-like, human-like) and Nationality as a between-subject factor. Recent studies showed that analyzing ordinal variables with metrics models can lead to distorted effect-size estimates, inflated error rates, and other problems [64, 65]. As an additional control analysis, we performed a Cumulative Link Mixed-Effect Model (CLMM) analysis to appropriately account for nonlinearities in the ordinal norming scale in both experiments [66]

## Results

### Explicit Measure of Attitudes Towards Robots

**Fig. 2:**
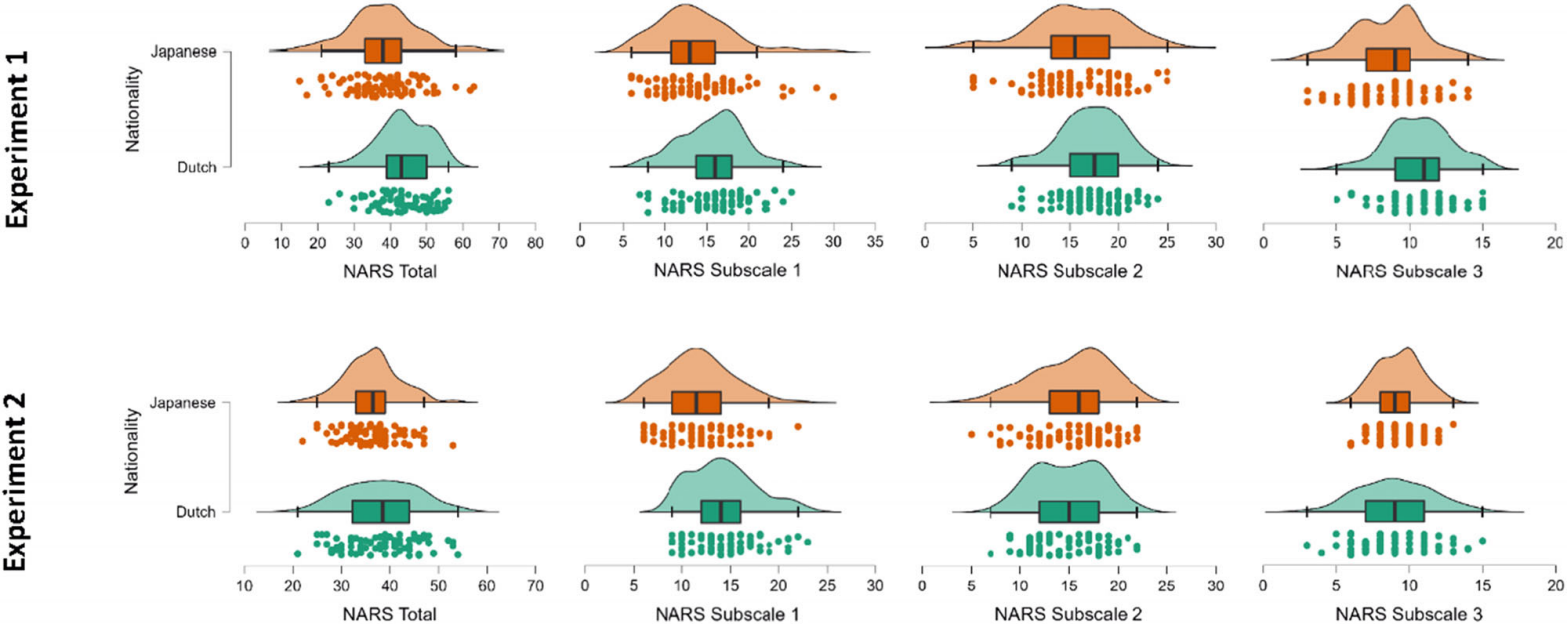
On the upper row, Japanese and Dutch comparison of the NARS total scores and NARS subscales for Experiment 1. Samples differ significantly on the NARS Subscale 3 (*p* = .053); on the bottom row, Japanese and Dutch comparison on the NARS total scores and NARS subscales for Experiment 2. Samples differ significantly on the NARS Subscale 1 (*p* < .001). The boxplots represent the mean score and standard deviation across all participants for the NARS subscales: for Experiment 1, NARS Subscale 3 scores ranged from 3 to 14 for the Japanese sample (Mean 8.59 ± 2.43) and from 5 to 15 for the Dutch sample (Mean 10.67 ± 2.18); for Experiment 2, NARS Subscale 1 score ranged from 6 to 22 for the Japanese sample (Mean 11.67 ± 3.44) and from 9 to 23 for the Dutch sample (Mean 14.21 ± 3.44)

In line with our hypothesis, we observed a small between-subject effect of nationality in Experiment 1 [F(1, 160) = 3.868, *p* = *.051*, η^2^p = 0.024], with a non-significant effect in the same direction for Experiment 2 [F(1, 154) = 2.995 *p* = *.086*, η^2^p = 0.019]. While participants from Japan (mean ± standard deviation (Experiment 1: 38.04 ± 9.34; Experiment 2: 36.30 ± 5.61) had a lower overall NARS score compared to Dutch participants (Experiment 1: 43.61 ± 7.18; Experiment 2: 36.14 ± 7.45), the nationality by subscale interaction was significant in both experiments [Experiment 1: F(1.93, 309.66) = 3.005, *p* = *.053*, η^2^p = 0.018; Experiment 2: F(1.93, 297.36) = 14.302, *p* < .001, η^2^p = 0.085]. In Experiment 1, post-hoc comparisons using the Holm test showed that this significance in explicit attitude is primarily driven by a difference on Subscale 3 of the NARS (*t*(160) = 1.271, *p* = *.045*, MD = 2.624, 95% CI [− 0.26, 4.46], d = 0.154), but not the other two scales, S1 (*t*(160) = 1.910*, p* = *.214*, 95% CI [− 0.83, 3.89]), and S2 (*t*(160) = − 0.017, *p* = *.986*, 95% CI [− 2.37, 2.35]). In Experiment 2, the interaction effect was explained by a difference on Subscale 1 of the NARS, (*t*(154) = 5.026, *p* < *.001*, MD = 2.538, 95% CI [1.04, 4.03]), but not the other two scales, S2 (*t*(154) = − 0.914, *p* = *.723*, 95% CI [− 1.77, 1.17]), and S3 (*t*(154) = − 0.482, *p* = *.723*, 95% CI [− 1.73, 1.24]). In other words, compared to Japanese participants, Dutch participants had a more negative attitude towards emotions in interaction with robots (Experiment 1) and towards social interactions with robots (Experiment 2) (Fig. 2). The results of the CLMM analysis confirmed that Japanese participants had a lower NARS scores than Dutch participants on Subscale 3 (Experiment 1: β = − 0.4470, SE = 0.112, * p* = *.021*) and Subscale 1 (Experiment 2: β = − 0.1334, SE = 0.058, *p* < *.001*).

### Implicit Measure of Attitudes Towards Robots and Avatars

In line with our prediction, the average *D*-scores were 0.26 (Experiment 1) and 0.27 (Experiment 2) for the robot IAT, and 0.31 (Experiment 1) and 0.32 (Experiment 2) in the avatar IAT respectively. Both scores were significantly different from 0.15 (Experiment 1: t = 7.207, *p* < *.001*, d = 0.563, 95% CI [0.122, 0.214]; t = 4.428, *p* =  < *.001*, d = 0.346, 95% CI [0.064, 0.167]; Experiment 2: t* = *6.950, *p* < *.001*, d = 0.556, 95% CI [0.122, 0.219]; t = 4.829, *p* =  < *.001*, d = 0.387, 95% CI [0.074, 0.387]), indicating a slight preference towards humans over artificial agents. Our second hypothesis was confirmed (H2a), as both Dutch and Japanese had a preference towards humans compared to Robots and Avatars. We found no significant difference between the D-score in the Avatar IAT compared to the Robot IAT (Experiment 1: t(162) = 1.785, *p* = *0.076*, 95% CI [− 0.005, 0.107]; Experiment 2: t(154) = 1.575, *p* = *0.117*, 95% CI [− 0.012, 0.103]).

**Fig. 3:**
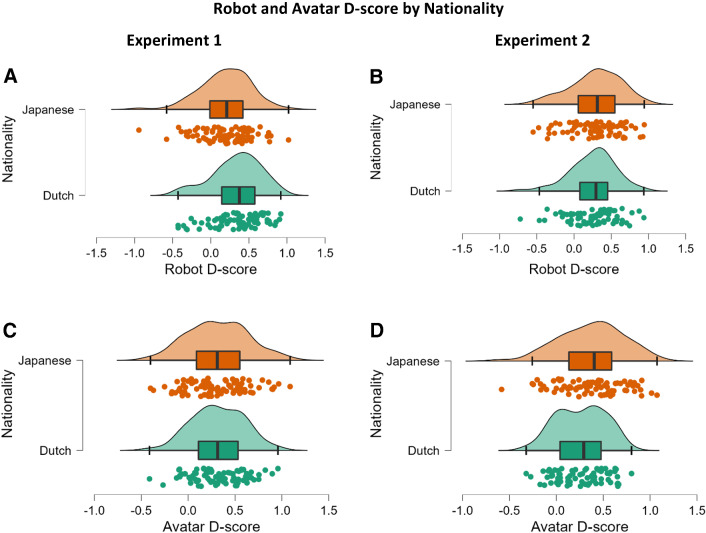
**a** D-score of the Robot IAT by Nationality in Experiment 1, ranging from − 0.94 to 1 for the Japanese sample (Mean 0.19 ± 0.32) and from -0.42 to 0.9 for the Dutch sample (Mean 0.33 ± 0.32); **b** D-score of the Robot IAT by Nationality in Experiment 2, ranging from − 0.55 to 1 for the Japanese sample (Mean 0.29 ± 0.33) and from − 0.72 to 0.93 for the Dutch sample (Mean 0. 25 ± 0.30); **c** D-score of the Avatar IAT by Nationality in Experiment 1, ranging from − 0.40 to 1 for the Japanese sample (Mean 0.31 ± 0.31) and from − 0.41 to 0.95 for the Dutch sample (Mean 0.31 ± 0.27); **d** D-score of the Avatar IAT by Nationality in Experiment 2, ranging from − 0.58 to 1 for the Japanese sample (Mean 0.37 ± 0.33) and from − 0.32 to 0.80 for the Dutch sample (Mean 0.26 ± 0.26)

In contrast to our prediction, there was no significant between-subject effect of Nationality (Experiment 1: F(1,160) = 0.738, *p* = *.39*; Experiment 2: F(1,154) = 3.224, *p* = *.07*) on the overall *D*-score. Japanese (Experiment 1: 0.19 ± 0.33—robot, 0.31 ± 0.31—avatar; Experiment 2: 0.29 ± 0.33—robot, 0.37 ± 0.33—avatar) and Dutch (Experiment 1: 0.33 ± 0.32—robot, 0.31 ± 0.27—avatar; Experiment 2: 0.25 ± 0.30—robot, 0.26 ± 0.26—avatar) participants did not differ in their implicit attitudes towards robots or avatars (Fig. 3).

### Implicit Measure of Attitudes Towards Different Types of Robots

Contrary to our hypothesis, we did not find a significant main effect of robots’ body types (Experiment 1: F(1,160) = 1.577, *p* = *.21*; Experiment 2: F(1,154) = 1,575, *p*=.216). No main effect of Nationality was found (Experiment 1: F(1,160) = 2.127, *p* = *.14*; Experiment 2: F(1,154) = 0.784, *p* = *.337*). An interaction effect between body type and nationality was observed only in Experiment 1 (F(1,160) = 5.979, *p* = *.016*, η^2^p = 0.036), but not in Experiment 2 (F(1,154) = 0.280, *p* = *.59*). Post-hoc comparison using Holm test for Experiment 1 revealed a slight preference for human-like robots only for the Dutch participants (*t*(160) =− 2.728, *p* = *.043*, MD = 0.128, 95% CI [− 0.080, 0.287], d =− 0.105), but not for the Japanese sample.

### Correlations Between Implicit and Explicit Attitudes

**Fig. 4:**
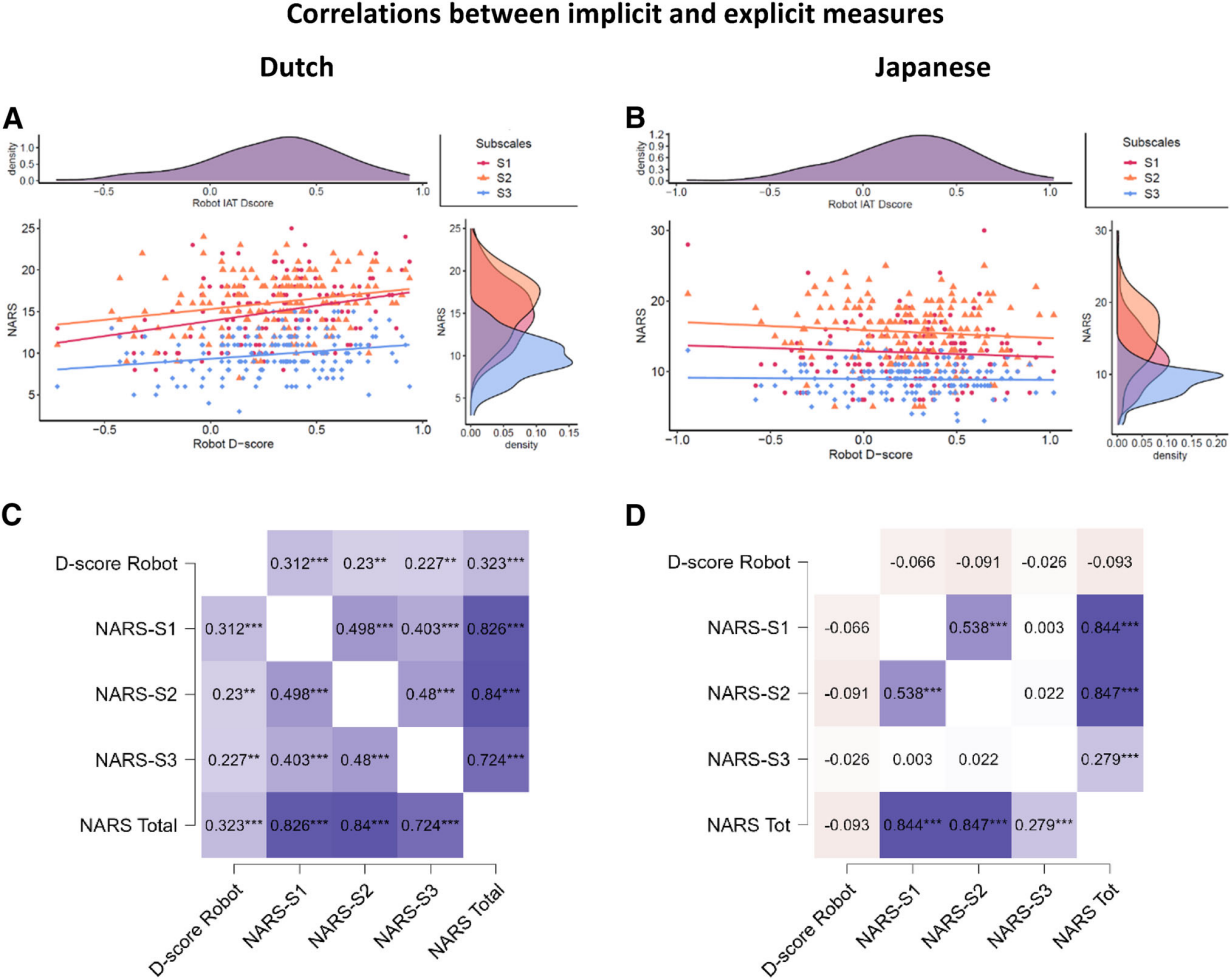
**A** correlation between Robot D-score and NARS Subscale 1 (*p* < .001), Subscale 2 (*p* = .004) and Subscale 3 (*p* = .004) for the Dutch sample in both experiments; **B** correlation between Robot D-score and NARS Subscale 1 (*p* = .0 = 407), Subscale 2 (*p* = .247) and Subscale 3 (*p* = .747) for the Japanese sample in both experiments; **C** correlations heatmap for Dutch sample in both experiments; **D** correlations heatmap for Japanese sample in both experiments. Significant correlations are flagged

As an exploratory analysis, we looked at the relationships between explicit and implicit attitudes for the Dutch and Japanese samples separately. Crucially, same-nationality samples from Experiment 1 and Experiment 2 were merged. Ten Pearson correlation tests between NARS and IAT were carried out for each nationality and tested against a Bonferroni-adjusted alpha level of 0.005 (0.05/10). Explicit and implicit measures of attitudes towards robots were correlated only for the Dutch sample (Fig. 4). All subscales of the NARS were positively correlated with the D-score in the Robot IAT (*r*(156) = 0.312, *p* < .001, two-tailed; *r*(156) = 0.230, *p* = .004, two-tailed; *r*(156) = 0.227, *p* =. 004, two-tailed). The results showed that the more the attitudes towards interactions, social influence, and emotions of robots were negative, the more Dutch participants showed an implicit preference towards humans. These correlations were not driven by a particular robot body type, as similar correlations were found for machine-like and human-like robots in both experiments (Fig. 4). No significant correlations were present in the Japanese sample between explicit and implicit measures in both experiments IAT (NARS-S1: *r*(160) = − 0.066, *p* = .407, two-tailed; NARS-S2: *r*(160) = − 0.091, *p* = .247, two-tailed; NARS-S3: *r*(160) = − 0.026, *p* = .747, two-tailed). Crucially, we found no correlation between the self-report measures and the D-score in the avatar task in both samples.

## Discussion

In the present study, we investigated the impact of individuals’cultural background on explicit *and* implicit attitudes towards robots and avatars. Using the NARS questionnaire and the IAT in Japanese and Dutch samples, we investigated the effect of cultural background and robots’ body type on these measures. Partly in line with our hypotheses, we found that the Japanese sample had more positive explicit attitudes towards robots compared to the Dutch, but no evidence of such a difference was found at the implicit level. As predicted by our second hypothesis, the implicit preference towards humans was moderate in both cultural groups, but in contrast to what we expected, neither culture nor robot embodiment influenced this preference. Together, these results suggest that cross-cultural differences in explicit attitudes do not generalize to implicit attitudes and that, also at the explicit level, these cultural discrepancies are not always comparable.

In both experiments, Dutch participants generally reported more negative explicit attitudes as measured by the NARS compared to Japanese participants. However, the cross-cultural discrepancies that were found are not the same across the two experiments. In Experiment 1 such difference was driven by subscale 3, namely *emotions in interaction with robots,* while in Experiment 2 it was driven by subscale 1, namely *situations of interaction* with robots. These subscales for instance measure how people would feel in actively engaging in a conversation with a robot (“I would feel paranoid talking with a robot”), or in interacting with a robot having emotions (“I feel comforted being with robots that have emotions”). When comparing our results to those of older studies, this finding is not surprising, since cross-cultural differences related to specific subscales have also been reported [39, 40]. Still, these previous outcomes diverge from ours as they report also differences between NARS Subscale 2, namely negative attitudes towards the social influence of robots. Our results, along with the previous findings, suggests that cross-cultural differences in explicit attitudes towards robot appear to exist, but on what precisely remains unclear. Despite the NARS has been widely used in human–robot interaction literature [37, 39, 53], the questionnaire may be a reliable measure of a general attitude towards the robot, but not sensitive enough to capture attitudes towards a specific aspect of human–robot interaction. The reliability of the NARS is indeed still debated: while some authors suggested that, when taken separately, the different factors of the NARS were not reliable enough [49], others reported high internal consistency for all the subscales of the NARS [40]. Regardless of the specific subscale in which we found a cross-cultural difference, the main discrepancy between our results and previous findings is about the *direction* of this difference. In prior experiments, it was the Japanese sample that had the most negative attitudes towards robots, in terms of social influence [39] and emotional interaction with robots [40] compared to other cultures including the Dutch one. The high exposure to robots in Japan is not negligible [67]. In both the aforementioned studies, the authors claimed that this high exposure may have given the Japanese population more realistic expectations of robots, with much awareness of their shortcomings [39, 40], which in turn may have been reflected in negative attitudes toward robots. Although we found an opposite trend, we believe that the explanation might in fact be the same: if on the one hand, the overexposure to robots can give the tools to be more critical and concerned about this technology [7], on the other it is undeniable that it can naturalize their presence in society. For this reason, the Japanese population may be more concerned about the social influence of robots, but this can coexist with their propensity for social interaction with robots found in the present study.

For the Dutch sample, contrary to a previous study [40], we found that participants had a generally more negative attitude towards robots compared to the Japanese one. Looking solely from the point of view of robot exposure, one could argue that the reduced frequency with which Dutch people experience human–robot interactions might enhance their concerns towards them. We believe that entirely explaining these results from the perspective of robot overexposure can be reductive, as our results resonate well also with the outcomes of recent works investigating Dutch attitudes towards the future [68, 69]. In terms of technology outlooks, Dutch respondents have proven to be particularly moderate: they expressed optimism about the overall impact of technology on society, but not without uneasiness towards specific technologies such as robotics and digitalization [68]. Especially regarding the intimate relationship with a robot, or the possibility for robots to make decisions about human lives, the vast majority of respondents expressed sincere concern and negative feelings [68, 69]. These results are directly in line with the negative attitudes towards social and emotional interactions with robots that Dutch participants reported in the present study.

Dutch and Japanese participants indicated more positive implicit attitudes towards humans both in the Robot and Avatar IAT. A similar conclusion has been reached by numerous previous studies, which have found more positive implicit attitudes towards humans compared to robots, assessed both with the IAT [53, 70, 71, 72] and with a different task [49]. A possible explanation lies in the assumption of similarity: the morphologic and biomechanics overlap is surely intensified with pictures of humans compared to robots, and this may have produced an ingroup bias already established in the IAT [73] and human–robot interaction literature [74]. If the morphologic overlap was the only explanation, we should have observed more positive attitudes towards avatars compared to robots. It is possible that categorizing robots as ingroup or outgroup might depend on factors that are not solely linked with morphological similarities. Supporting this position, a study found negative implicit attitudes even for hyper-realistic robots [75]. As much as we can create robots similar to humans, they will still seize a plethora of unique features that will always make them fundamentally different from us, a distinct social category [74].

Along with the preferences towards humans in both cultures, we still expected Japanese participants to have slightly more positive implicit attitudes towards robots and avatars, with a significant decrease in the average D-score compared to the Dutch one. The results do not support our hypothesis, since the differences in D-score between Japanese and Dutch participants are negligible in both tasks. This lack of effect of the nationality in the implicit attitudes towards robots, but not in the explicit ones, is directly in line with previous findings: some authors found that Japanese participants expressed more positive explicit attitudes towards robots compared to the US sample, but these differences were absent at the implicit level [36]. This conflicted association between explicit and implicit measures in the Japanese sample, combined with a non-significant correlation amongst the measures, is not new in the literature and it could mean that people implicitly have different opinions about robots than they explicitly want to or can reveal [76]. A desire to conform can lead to a self-presentational bias [9]. Participants choose to present themselves to others which may not accurately reflect their attitudes and dispositions owing to concerns about social desirability [9, 77]. Cross-cultural differences might be at play in this process. Japan, for instance, considers itself a robot-friendly country [78]. This does not necessarily imply that, at the individual level, Japanese participants all would have positive implicit attitudes, but might impact the way participants chose to present themselves [36]

Another possible explanation resonates with MacDormand and colleagues' findings, as this ambivalence may partially depend on a mismatch between the robot mental representation that each participant had active while filling NARS and the actual robots shown during the IAT [35]. In fact, people’s assumptions about robots widely range from humanoid to laboratory robots, industrial robots, and so on [79]. This might influence implicit measures even though Japanese participants rationally reported willingness to interact with the robots. The mental representation of a robot that participants have while filling out the self-report questionnaire might be very different from the actual robots shown in the pictorial IAT. This speculation, however, holds only for the Japanese participants, as for Dutch participants explicit and implicit attitudes were positively correlated. A more negative explicit attitude towards interactions and the social influence of robots was correlated with an implicit preference towards humans.

Implicit and explicit measures have often been seen as discordant with each other [80]. However, it has been shown that spontaneity—not engaging in the effortful process of retrieving recently formed representations from memory while completing self-report questionnaires—increases the correlation between them [81]. As Japanese people are highly exposed to robots, they could also retrieve more representations while filling self-report questionnaires; on the contrary, Dutch people might rely on fewer mental images of robots and answer more spontaneously. Due to the mixed results in this study, it remains unclear to which degree implicit and explicit measures are correlated. It is important to underline that a lack of correlation may depend on the limitations of the implicit task in itself. The IAT has in fact received several criticisms from the literature. First, some authors proposed that the test might measure mere familiarity [68], but it has been shown that the stimulus familiarity did not affect the automatic race associations at the IAT [69]. More importantly, the test foresees an opposition between two concepts, postulating a symmetrical relation between them. Following the reasoning of Spatola and Wudarczyk, robots are not necessarily antagonist categories [49]. The authors proposed a new implicit measure lacking this bi-dimensional categorization in which there is a priming of a semantic link that is independent of the other category. In their task, human and robot are never explicitly opposed as in the standard IAT. Future studies should investigate implicit measures by the means of this new task, perhaps comparing it with the classic IAT. At the same time, the mismatch between explicit and implicit measures found in the present study is in line with the recent work in the field of Human–Robot Interaction. Li and colleagues examined the mind perception of robots using a manipulated version of the IAT (MP-IAT) and an explicit mind perception rating [82]. As there was no correlation between constructs, the authors suggested that implicit and explicit measures might be related but potentially independent concepts that require independent assessment. Acknowledging the limitations of the testing method, we are still keen to consider the IAT as a useful tool in Human–Robot Interaction to understand human perception of robots.

How culture influences the preference toward human-like or machine-like robots remains elusive. Only in one experiment we find that Dutch participants have a slight preference for human-like robots compared to machine-like ones compared to Japanese participants. This is inconsistent with previous studies that found a preference towards machine-like robots [24, 28, 29, 45], but still in line with several works where people reported preferring human-like robots [20, 83, 84]. The majority of the studies that found a preference towards machine-like robots were in fact comparing them with hyper-realistic humanoid robots [29, 45], which can be perceived as more threatening [70]. Since Japanese people have previously shown a reluctance towards hyper-realistic androids [45], we chose to not use pictures of realistic humanoids, but rather of robots that have some characteristics attributable to humans (limbs, head, face). Given the lack of a difference even with this foresight, we wondered whether this appearance of the robots has been actually perceived as human-like or machine-like at all. In fact, while some studies would agree with us considering this shape as human-like [8], others used similar robots as machine-like counterparts for hyper-realistic humanoids [75]. We encourage performing a pre-test validation of the robot stimuli using the ABOT database [60], which could help to categorize the robots’ embodiment in a standardized manner across papers and disciplines.

Another explanation might lie in the importance of the context for the preferred appearance of robots. There is a general consensus that the preference for a specific robot appearance also depends on the social domain in which it will be used [16]. So far, the nature of this context-dependency remains unknown. It has been shown that the body type preference for household robots can be humanoid but not hyper-realistic [84], while another study found that human appearance was strongly undesirable and a domestic robot should look like a small machine-like robot [28]. Other authors suggested that this preference is really affected by individual differences, as some people preferred tall and humanoid domestic robots, while others had a preference towards mechanical-looking robots [8]. In terms of reliance, literature shows that humanoid robots might be more appropriate to delegate tasks, while machine-likes robots still make the participants feel more responsible for the task [24]. This huge body of work suggests that it’s dangerous to abstract the preference toward a particular robot body type from the specific social context in which it will be used. Future research on both implicit and explicit attitudes towards robots should frame the experiment within a specific context of use, perhaps looking at the implicit association between different robot embodiments and contextual tasks they might take over.

## Limitations and Future Directions

A limitation of our research is the lack of assessment for previous experiences with robots. Research has shown that personal experiences with artificial agents, be it embodied interactions or exposure through the arts, are crucial in shaping our attitudes towards them and our willingness to interact with them [40, 67]. These experiences can steer a mental representation of the artificial agent that, in turn, might influence our spontaneity in filling in self-report questionnaires [85] but also modulate our implicit attitudes towards the agents themselves [86]. Future research should strategically control this. In conjunction with experience, beliefs, and other cognitive factors, the shape and form of the artificial agent determine how the observer evaluates this agent [11]. In the present study, we used machine-like and human-like robots. It remains unclear to what extent these labels represent prototypical machine-like and human-like robots as the definition of machine-like and human-like robots or related labels is extremely vague in the literature. In order to fully understand the interplay between attitude and shape, form, embodiment, a clearer consistency in the definition of the different types of robots (e.g., machine-like, human-like, humanoid) is warranted.

In the current study, explicit and implicit measures were assessed together. As the relationship between those measures is still unclear, it remains to be understood whether one could influence the other and vice versa. Future studies could investigate these attitudes at separate moments to control for this potential effect. It is worth mentioning that both our implicit and explicit measures are negatively framed: the IAT test implies a conceptual opposition between artificial agents and humans, and in the NARS two out of three subscales contain items negatively framed. Considering the acquiescence bias, which occurs when people tend to agree with statements without regard for their actual content [87], it is very likely that positively framed scales could elicit different responses in the participants. Salazar (2015) also founds a greater tendency for the items in a positive questionnaire to show results associated with the directionality of the [88]. However, in absence of cross-cultural literature on this matter, it is difficult to speculate about the effect of positively vs negatively framed scales on the magnitude of the cultural differences. Human–robot interaction literature would benefit from comparing positively and negatively framed questionnaires to investigate their sensitivity in capturing cultural differences. Critically, despite online experiments represent a useful and valid tool [89], they also come with less control by the researchers. Future research should also test this in different context beyond online experiments. Finally, we encourage a much more extensive inclusion of cultural samples in order to avoid over-and under-representation. A review by Lim and colleagues call for investigations beyond the West/European-East/Asian dichotomy, within the framework of the individualism-collectivism dimension to examine whether and under which circumstances certain cultures could be more flexible in developing positive perceptions and attitudes towards robots [7]. In this regard, the recent study by Marchesi and colleagues showed that the cultural values expressed by the individualist-collectivist dimension have a greater effect than nationality on our implicit attitudes toward robots [48]. This highlights the need to investigate cross-cultural differences through this still underexplored lens.

In conclusion, our study expanded the knowledge about the effect of agents’ embodiment and of the users’ cultural background on the attitudes towards robots and avatars, but also about the relationship between explicit and implicit measures to assess human attitudes towards artificial agents. Collectively, our results suggest that cross-cultural differences in the explicit attitudes towards robots exist, but these are not necessarily accompanied by implicit differences.

## Supplementary Information

Below is the link to the electronic supplementary material.

Supplementary file1 (DOCX 92 KB)

## Footnotes

1 Because of the large number of excluded participants due to incomplete data, a summary table of the demographic information can be found in Supplementary Table 1c.
