# Supplementary material for: A Cross-Cultural Comparison on Implicit and Explicit Attitudes Towards Artificial Agents
Source: Int J Soc Robot. 2022 Sep 28;15(8):1439–55. doi: 10.1007/s12369-022-00917-7 (PMC10465401; doi:10.1007/s12369-022-00917-7)
Supplement: Supplementary file 1 — Supplementary file1 (DOCX 92 KB) [file 12369_2022_917_MOESM1_ESM.docx]

# Supplementary material

## Title

Implicit and explicit attitudes towards artificial agents: a cross-cultural comparison

## Journal name

International Journal of Social Robotics

## Authors

Fabiola Diana^1,2^, Misako Kawahara^3^, Isabella Saccardi^4^, Ruud Hortensius^5^, Akihiro Tanaka^3^, Mariska E. Kret^1,2^

## Corresponding Author

Fabiola Diana, [d.fabiola@fsw.leidenuniv.nl](mailto:d.fabiola@fsw.leidenuniv.nl), Comparative Psychology and Affective Neuroscience Lab, Cognitive Psychology Unit, Leiden University, Netherlands

## Tables

|  |  | Mean Age | Graduated | Under graduated | Females | Males |
| --- | --- | --- | --- | --- | --- | --- |
| Experiment 1 | Dutch | 19.75(±2.38) | 86,2% | 13,7% | 80,7% | 19,2% |
|  | Japanese | 25,88(±3.67) | 51,1% | 48,8% | 50% | 50% |
| Experiment 2 | Dutch | 42.65 (±5.85) | 82% | 17,9% | 42,3% | 57,6% |
|  | Japanese | 41.29 (±9.60) | 76,9% | 23% | 52,5% | 47,4% |

Supplementary Table 1a: Additional demographic information for the included samples in the two experiments

| Level of education | ISCED | Dutch Sample (1) | Japanese Sample (1) | Dutch Sample (2) | Japanese Sample (2) |
| --- | --- | --- | --- | --- | --- |
| Primary Education | 1 | 0 % | 0 % | 1,28 % | 0 % |
| Lower secondary education | 2 | 2,50 % | 7,14 % | 10,25 % | 8,97 % |
| Upper secondary education | 3 | 6,25 % | 4,76 % | 2,56 % | 8,97 % |
| Post-secondary education | 4 | 77,50 % | 36,90 % | 3,84 % | 3,84% |
| Short-cycle tertiary education | 5 | 0 % | 0 % | 0 % | 0 % |
| Bachelor’s or equivalent | 6 | 10 % | 46,42 % | 44,87 % | 62,82 % |
| Master’s or equivalent | 7 | 2,50 % | 3,5 % | 30,76 % | 10,25 % |
| Doctorate or equivalent | 8 | 1,25 % | 1,19 % | 5,12 % | 3,84 % |

Supplementary Table 1b: Dutch and Japanese participants' level of education in percentage, based on the International Standard Classification of Education (ISCED) of UNESCO

|  |  | Mean Age | Graduated | Under graduated | Females | Males |
| --- | --- | --- | --- | --- | --- | --- |
| Experiment 1 | Dutch | 23.23(±9.33) | 94,7% | 5,2% | 65,7% | 34,2% |
|  | Japanese | 27.05(±4.35) | 54,2% | 45,7% | 60,7% | 39,2% |
| Experiment 2 | Dutch | 42.54(±6.17) | 83,3% | 16,1% | 51,6% | 45,6% |
|  | Japanese | 38.60(±10.39) | 64,8% | 35,1% | 56,3% | 43,6% |

Supplementary Table 1c: Demographic information of the participants excluded because of incomplete data

| Robot name | Human-likeness |
| --- | --- |
| Silverlit | 41.55 |
| Darwin | 40.04 |
| Tetrix | 31.01 |
| Manoi AT01 Type-C body | 41.55 |
| Wow Wee Robosapien | 38.95 |
| Pepper | 28.64 |
| Thespian | 47.27 |
| QurRong | 34.32 |
| NAO | 38.42 |
| HRP-4 | 44.22 |
| Aelos S1 | 41.13 |
| NARJOG ViVi | 29.39 |
| Leju | 23.93 |
| Romeo | 47.27 |
| OSP | 28.91 |
| DJI RoboMaster S1 | 0.97 |
| Jalshree | 2.05 |
| Niryo | 0 |
| Vector | 13.87 |
| Cozmo | 13.87 |
| Mi Robot Builder Rover | 13.88 |
| DIY Robotic arm | 0 |
| MiP | 19.12 |
| Mazebreaker | 16.04 |
| Cady Wile | 17.02 |
| Miko 3 | 14.25 |
| Cruzr | 11.50 |
| Zenbo Junior II | 15.06 |

Supplementary Table 2: human-likeness score of the robots calculated using the ABOT database (http://www.abotdatabase.info/)

| Japanese unpleasant words | Dutch unpleasant words |
| --- | --- |
| 苦痛 | Ondraaglike pijn |
| 危険な | Gevaarlijk |
| あてにならない | Onbetrouwbaar |
| 怖い | Verschrikkelijk |
| 不愉快な | Vreselijk |
| 卑劣な | Smerig |
| 害悪 | Kwaadaardig |
| ひどい | Afschuwelijk |
| 失敗 | Falen |
| 死 | Dood |
| 傷ついた | Pijn |
| 殺人者 | Moordenaar |

Supplementary Table 3: list of Japanese and Dutch unpleasant word used in the Implicit Association Test (IAT) task

| Japanese pleasant words | Dutch pleasant words |
| --- | --- |
| 喜び | Vreugde |
| 愛情 | Liefde |
| 平和 | Vrede |
| 信頼できる | Gelacht |
| 愉快 | Genot |
| 頼りになる | Glorieus |
| 幸せな | Blij |
| 協力的な | Prachtig |
| 成功 | Success |
| 親切 | Vriendelijkheid |

Supplementary Table 4: list of Japanese and Dutch pleasant word used in the Implicit Association Test (IAT) task

## Supplementary Analysis

### Experiment 1 – Linear regressions to control for Age

We performed three linear regressions with age as a covariate and Nationality as a factor to predict the dependent variables central to the hypothesis (NARS score, Robot D-score, Avatar D-score). No issue of multicollinearity was detected. Age did not significantly predict the explicit attitude towards robot (*t*(163)=-1.640, *p=.103*, 95% CI [-0.75, 0.70]), nor the implicit attitude towards robot (*t*(163)=-0.064, *p=.949*, 95% CI [-0.017, 0.016]), and avatar (*t*(163)=-0.622, *p=.535*, 95% CI [-0.020, 0.010]).

### Experiment 1 – Effect of Age and Level of education

We found no significant effect of Age on the explicit attitudes towards robot and avatar (F(1, 160) = 3.099, *p=.080*, η²p = 0.019), nor on the implicit attitudes (F(1, 160) = 0.020, *p=.889*, η²p = 0.0001). Crucially, we did not found an effect of Age in the implicit attitude towards different types of robots (F(1, 160) = 0.0003, *p=.985*, η²p <.0001). We found no significant effect of Level of Education on the explicit attitude towards robot and avatar (F(1, 160) = 0.847, *p=.359*, η²p = 0.005), nor on the implicit attitudes (F(1, 160) = 3.013, *p=.085*, η²p = 0.018). However, we did find a between-subject effect of level of education on the implicit attitude towards robot (F(1, 160) = 5.925, *p=.016*, η²p = 0.036), but there was no significant interaction effect between type of robot and level of education (F(1, 160) = 0.844, *p=.360*, η²p = 0.005), (see Supplementary Figure 1).

| **Robot Types’ D-scores plot by Level of Education** |
| --- |
| 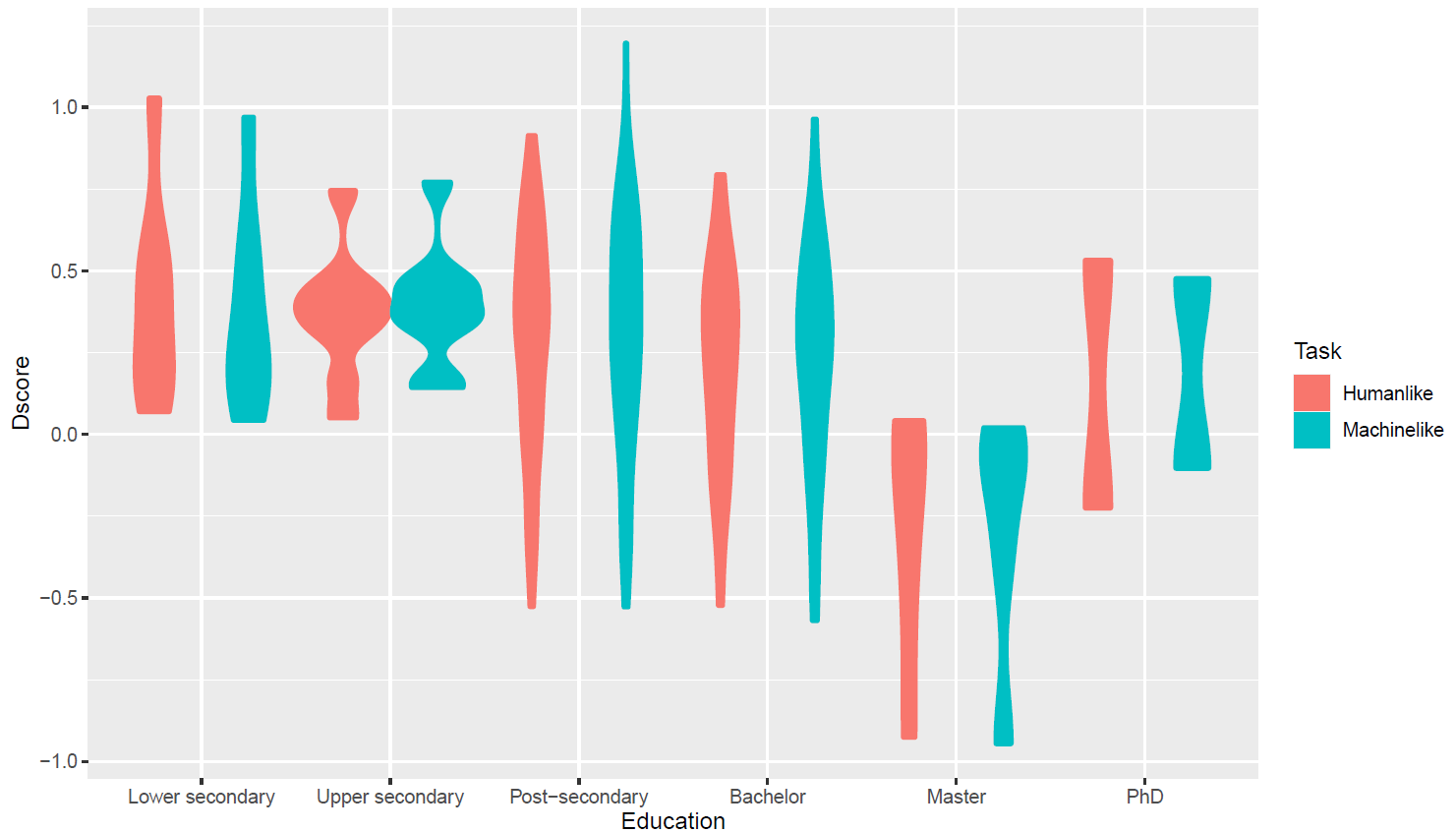  Supplementary figure 1: human-like and machine-like robot D-scores plot by Level of Education |

## Correlations

### Correlations between NARS and Type of Robots

We found correlations between NARS subscales and the D-score in the Robot IAT only for the Dutch participants. To verify that these correlations were not driven by a particular robot body type, we carried out fifteen Pearson correlations tests between NARS, human-like D-score, and machine-like D-score tested against a Bonferroni-adjusted alpha level of 0.003 (0.05/15). Our results indicate that the correlations between NARS subscales and Robot IAT D-score were not driven by a particular body type as respective correlations were found both for machine-like (NARS-S1: r=0.319, p<.001; NARS-S3: r=0.240, p=.002) and human-like D scores (NARS-S1: r=0.319, p<.001; NARS-S2: r=0.247, p=.002, NARS-S3: r=0.236, p=.003). The only exception is the correlation between machine-like D-score and NARS-S2, whose p-value does not reach the Bonferroni-adjusted significance (r=0.214, p=.007).

### Fitting Cumulative Linear Mixed Model based on Taylor et al., (2021)

Studies that provide norms of Likert ratings typically report per-item summary statistics. Traditionally, these summary statistics comprise the mean and the standard deviation (SD) of the ratings, and the number of observations. Such summary statistics can preserve the rank order of items, but provide distorted estimates of the relative distances between items because of the ordinal nature of Likert ratings. Inter-item relations in such ordinal scales can be more appropriately modeled by cumulative-link mixed-effects models (CLMMs).

Although the generative Bayesian model can afford rich posterior distributions, we fitted an equal-variance model with maximum likelihood as we were only interested in estimating per-item latent means. The authors estimated r=.997 correlation between the two approaches, meaning the simpler equal variance model and the Bayesian model provide extremely similar estimations and equal variance models can generally be used to estimate latent means without any great loss inaccuracy. In addition, despite CLMM providing more accurate estimates for norming studies, the authors encourage reporting them *in addition* to more traditional measures like Likert means and SDs, rather than *instead* of them. This is important to ensure that results are still comparable with the existing dataset reporting only mean and SDs.

All CLMM models were fitted using the *ordinal* package in R v4.1.2 (R Core Team, 2016), using the script released by Taylor et al., (2021). For Experiment 1, we fitted a CLMM with Nationality, Nationality*NARS Subscale, Age and Level of Education as Fixed effect, Participants and Subscales as Random Effect, and the NARS scores as Dependent variable. Results go in the same direction as in the rm ANOVA. We found a significant effect of NARS Subscales Nationality*Subscales interaction (β = -0.1345, SE = 0.058, p=.020). We did not found a significant effect of Nationality (β = -0.04635, SE = 0.187, p=.804), Age (β = -0.03320, SE = 0.018, p=.066) and Level of Education (β = 0.7232, SE = 0.048, p=.137). For Experiment 2, we fitted a CLMM model with Nationality, Nationality*NARS Subscale as Fixed effect, Participants and Subscales as Random Effect, and the NARS scores as Dependent variable. Results go in the same direction as in the rm ANOVA. We found a significant effect of Nationality (β = -0.7556, SE = 0.136, p<.001) and Nationality*Subscales interaction (β = 0.3294, SE = 0.059, p<.001). Crucially a between-subject effect of Nationality was confirmed for Experiment 2 but not for Experiment 1 corroborating the concerns related to the use of metric analyzes on ordinal variables (Liddel & Kruschke, 2018; Taylor et al., 2021).

# McDonald’s Omega calculation

We estimated the Omega coefficient (ωu) following the guidelines and the scripts provided by Flora (2020). For both experiments, we estimated the coefficients separately for each subscale and each sample.

## **Experiment 1 – Dutch sample**

### NARS Susbscale 1

We fitted a one-factor model for the Negative Attitudes towards Situations and Interactions with Robots including all the items in the aforementioned subscale of the NARS questionnaire as fixed effects. With the residuals command on the fitted model, we found a notable residual correlation between items 7 and 12 (.098) and 10 and 12 (.095). Therefore, we respecified the one-factor model for the Subscale 1 items, but now include a new line to specify the free error covariance between items 7 and 12 and items 10 and 12. Based on the Anova function between the two models, adding the free error covariance between items did not improve significantly the fitting of the model (p=.218). Therefore, we report the reliability based on the model that is not accounting for free error covariance. The reliability of NARS Subscale 1 for Experiment 1 was **ωu = 0.711**.

### NARS Subscale 2

We fitted a one-factor model for the Negative Attitudes toward Social Influence of Robots including all the items in the aforementioned subscale of the NARS questionnaire as fixed effects. The residuals analysis did not reveal any notable correlations between the items. The reliability of NARS Subscale 2 for Experiment 1 was **ωu = 0.694**.

### NARS Subscale 3

We fitted a one-factor model for the Negative Attitudes toward Emotions in Interaction with Robots including all the items in the aforementioned subscale of the NARS questionnaire as fixed effects. The residuals analysis did not reveal any notable correlations between the items. The reliability of NARS Subscale 2 for Experiment 1 was **ωu = 0.643**.

## **Experiment 1 – Japanese sample**

### NARS Susbscale 1

We fitted a one-factor model for the Negative Attitudes towards Situations and Interactions with Robots including all the items in the aforementioned subscale of the NARS questionnaire as fixed effects. With the residuals command on the fitted model, we found a notable residual correlation between items 4 and 10 (.103). Therefore, we respecified the one-factor model for the Subscale 1 items, but now include a new line to specify the free error covariance between items 4 and 10. Based on the Anova function between the two models, adding the free error covariance between items did improve significantly the fitting of the model (p<.05). Therefore, we report the reliability based on the model that is accounting for free error covariance. The reliability of NARS Subscale 1 for Experiment 1 was **ωu = 0.868**.

### NARS Subscale 2

We fitted a one-factor model for the Negative Attitudes toward Social Influence of Robots including all the items in the aforementioned subscale of the NARS questionnaire as fixed effects. The residuals analysis did not reveal any notable correlations between the items. The reliability of NARS Subscale 2 for Experiment 1 was **ωu = 0.859**.

### NARS Subscale 3

We fitted a one-factor model (j_mod3f) for the Negative Attitudes toward Emotions in Interaction with Robots including all the items in the aforementioned subscale of the NARS questionnaire as fixed effects. The residuals analysis did not reveal any notable correlations between the items. The reliability of NARS Subscale 2 for Experiment 1 was **ωu = 0.807**.

## **Experiment 2 – Dutch**

### NARS Susbscale 1

We fitted a one-factor model for the Negative Attitudes towards Situations and Interactions with Robots including all the items in the aforementioned subscale of the NARS questionnaire as fixed effects. With the residuals command on the fitted model, we found a notable residual correlation between items 4 and 7 (.111), 7 and 9 (.112), and 7 and 10 (-.133). Therefore, we respecified the one-factor model for the Subscale 1 items, but now include a new line to specify the free error covariance between items 4 and 7, 7 and 9, and 7 and 10. Based on the Anova function between the two models, adding the free error covariance between items did not improve significantly the fitting of the model (p=.136). Therefore, we report the reliability based on the model that is not accounting for free error covariance. The reliability of NARS Subscale 1 for Experiment 2 was **ωu = 0.683**.

### NARS Subscale 2

We fitted a one-factor model for the Negative Attitudes toward Social Influence of Robots including all the items in the aforementioned subscale of the NARS questionnaire as fixed effects. With the residuals command on the fitted model, we found a notable residual correlation between items 1 and 14 (.155), items 11 and 14 (.209), and items 13 and 14 (.091). Therefore, we respecified the one-factor model for the Subscale 1 items, Therefore, we respecified the one-factor model for the Subscale 1 items, but now include a new line to specify the free error covariance between items 1 and 14, items 11 and 14, and items 13 and 14. Based on the Anova function between the two models, adding the free error covariance between items improved significantly the fitting of the model (p<.005). Therefore, we report the reliability calculated by accounting for the free error covariance. The reliability of NARS Subscale 2 for Experiment 2 was **ωu = 0.681**.

### NARS Subscale 3

We fitted a one-factor model for the Negative Attitudes toward Emotions in Interaction with Robots including all the items in the aforementioned subscale of the NARS questionnaire as fixed effects. The residuals analysis did not reveal any notable correlations between the items. The reliability of NARS Subscale 2 for Experiment 1 was **ωu = 0.784**.

## **Experiment 2 – Japanese**

### NARS Susbscale 1

We fitted a one-factor model (j_mod1f-2) for the Negative Attitudes towards Situations and Interactions with Robots including all the items in the aforementioned subscale of the NARS questionnaire as fixed effects. With the residuals command on the fitted model, we found a notable residual correlation between items 4 and 7 (.204), and 4 and 10 (.138). Therefore, we respecified the one-factor model for the Subscale 1 items, but now include a new line to specify the free error covariance between items 4 and 10 (j_mod1fR-2). Based on the Anova function between the two models, adding the free error covariance between items improved significantly the fitting of the model (p<.005). Therefore, we report the reliability calculated by accounting for the free error covariance. The reliability of NARS Subscale 2 for Experiment 2 was **ωu = 0.703**.

### NARS Subscale 2

We fitted a one-factor model for the Negative Attitudes toward Social Influence of Robots including all the items in the aforementioned subscale of the NARS questionnaire as fixed effects. With the residuals command on the fitted model, we found a notable residual correlation between items 1 and 2 (.108), items 1 and 13 (-.100), items 1 and 14 (-.116), items 11 and 13 (.142), and items 11 and 14 (.156). Therefore, we respecified the one-factor model for the Subscale 1 items, but now include a new line to specify the free error covariance between the aforementioned items dyads. Based on the Anova function between the two models, adding the free error covariance between items improved significantly the fitting of the model (p<.001). Therefore, we report the reliability calculated by accounting for the free error covariance. The reliability of NARS Subscale 2 for Experiment 2 was **ωu = 0.760**.

### NARS Subscale 3

We fitted a one-factor model for the Negative Attitudes toward Emotions in Interaction with Robots including all the items in the aforementioned subscale of the NARS questionnaire as fixed effects. The residuals analysis did not reveal any notable correlations between the items. The reliability of NARS Subscale 2 for Experiment 1 was **ωu = 0.625**.
